# Quality-of-life scale machine learning approach to predict immunotherapy response in patients with advanced non-small cell lung cancer

**DOI:** 10.3389/fimmu.2025.1600265

**Published:** 2025-07-18

**Authors:** Juanyan Shen, Junliang Ma, Shaolin Chen, Su-Han Jin, Junzhu Xu, Qisha Li, Chi Zhang, Xiaojing Tian, Xiaofei Chen, Fangya Tan, Markus Hecht, Benjamin Frey, Udo S. Gaipl, Hu Ma, Jian-Guo Zhou

**Affiliations:** ^1^ Department of Oncology, The Second Affiliated Hospital of Zunyi Medical University, Zunyi, China; ^2^ Department of Thoracic Surgery, The Third Affiliated Hospital of Zunyi Medical University, The First People's Hospital of Zunyi, Zunyi, Guizhou, China; ^3^ Department of Thoracic Surgery, Affiliated Hospital of Zunyi Medical University, Zunyi, Guizhou, China; ^4^ Nursing Department, Affiliated Hospital of Zunyi Medical University, Zunyi, Guizhou, China; ^5^ Department of Orthodontics, Affiliated Stomatological Hospital of Zunyi Medical University, Zunyi, China; ^6^ Oncology Biometrics, AstraZeneca, Gaithersburg, MD, United States; ^7^ Harrisburg University of Science and Technology, Harrisburg, PA, United States; ^8^ Department of Radiotherapy and Radiation Oncology, Saarland University Medical Center, Homburg, Germany; ^9^ Translational Radiobiology, Department of Radiation Oncology, Universitätsklinikum Erlangen, Friedrich-Alexander-Universität Erlangen-Nürnberg, Erlangen, Germany; ^10^ Comprehensive Cancer Center Erlangen-Europäische Metropolregion Nürnberg (EMN), Erlangen, Germany; ^11^ Friedrich-Alexander-Universität (FAU) Profile Center Immunomedicine (FAU I-MED), Friedrich-Alexander- Universität Erlangen-Nürnberg, Erlangen, Germany; ^12^ Department of Biostat and Programming, Sanofi, Bridgewater, NJ, United States

**Keywords:** quality of life, consensus clustering, atezolizumab, NSCLC, overall survival

## Abstract

**Background:**

Despite immune checkpoint inhibitors(ICIs) significantly improve clinical outcomes in patients with advanced non-small cell lung cancer (aNSCLC), disease progression is inevitable. A diverse patient-reported Quality-of-life(QoL) scales were used to predict outcomes for aNSCLC patients with atezolizumab using machine learning.

**Materials and Methods:**

This study analyzed the association between baseline QoL and clinical outcomes in aNSCLC patients with atezolizumab in 4 randomized clinical trials: the IMpower150 study (discovery cohort), the BIRCH, OAK and POPLAR study (validation cohorts). We identified quality of life subtypes (QoLS) by consensus clustering in the discovery cohort and predicted them in external validated cohorts.

**Results:**

We identified QoLS1 and QoLS2 via consensus clustering in the discovery cohort. Compared with QoLS1, QoLS2 was associated with significantly worse survival outcomes, including a shorter median overall survival (OS: 13.14 *vs*. 21.42 months, hazard ratio (HR) 2.07, 95% CI: 1.64 to 2.62; *p* < 0.0001) and progression-free survival (PFS: 5.7 *vs*. 8.3 months, HR 1.69, 95% CI 1.42 to 2.04; *p* < 0.0001). QoLS2 also was associated with lower clinical benefit rate (57% *vs*. 68%, *p* = 0.0027). In external cohorts, QoLS2 was consistently associated with unfavorable OS (*p* < 0.0001). Notably, QoLS1 was a positive predictive biomarker for atezolizumab efficacy: patients in QoLS1 group derived greater survival benefit from ICIs versus chemotherapy (IMpower150, *p* = 0.04; OAK+POPLAR, *p* = 0.007), while patients in QoLS2 showed no significant treatment benefit.

**Conclusions:**

Our study demonstrated the potential of integrative machine learning in effectively analyzing baseline QoL and predicting clinical outcomes in aNSCLC patients undergoing atezolizumab immunotherapy.

## 1 Introduction

Immunotherapy with immune checkpoint inhibitors (ICIs) has revolutionized the treatment landscape for various cancers, including advanced non-small cell lung cancer (aNSCLC) (1–3). However, despite impressive responses in some individuals, a significant proportion of patients do not achieve sustained disease control and may experience primary or acquired resistance (4, 5). Given the substantial financial burden (6) and potential for severe immune-related adverse events with ICIs (7), there is a critical need to identify patients most likely to benefit and to optimize treatment selection for precision oncology (8). Therefore, the development of reliable and validated biomarkers to predict response to anti-PD-1/PD-L1 immunotherapy has emerged as a central focus in oncological immunology research. To date, biomarkers related to immunotherapy have concentrated on molecular analyses of the tumor immune microenvironment, including the assessment of PD-L1 (9) and TMB (10). However, such biomarkers face barriers that hinder their widespread clinical implementation, including tumor tissue acquisition is challenging, with inconsistent detection platforms, complex procedures, and high costs (11–13). This highlights an urgent clinical need for easily accessible, inexpensive, non-invasive, and widely available predictive markers (14, 15). Patient-reported outcomes(PROs), encompassing quality of life (QoL), physical symptoms, founction status, and psychological distress, are significant patient-centered outcomes for cancer patients (16). PROs offer a unique, non-invasive, tailored assessment of a patient’s QoL and symptoms, directly reported by the patient and easily administered in clinical settings. Previous studies have shown that baseline PROs, such as lower QoL (17), emotional distress (18), and higher symptom burden (19) are associated with a shorter overall survival in different cancer groups. Indeed, PROs have been established as independent predictors for cancer outcomes (20), providing valuable information that can complement or extend the predictive power of traditional clinical markers like serum tumor markers (21) and The Eastern Cooperative Oncology Group Performance Status(ECOG-PS) (22). For instance, pretreatment PROs were found to be independent prognostic markers for progression-free survival (PFS) in patients diagnosed with hormone receptor-positive, human epidermal growth factor receptor 2-negative (HR+/HER2-) advanced breast cancer treated with abemaciclib (22). Nevertheless, the consistency of PROs’ prognostic significance across different cancer types and treatment modalities, particularly in the context of immunotherapy, remains an area of active investigation. Furthermore, their potential to serve as predictive markers for treatment efficacy, specifically identifying patients who will derive differential benefit from immunotherapies alongside prognosis, is not yet fully characterized (23).

Building upon the established prognostic value of PROs, this study aimed to investigate whether distinct patient subtypes, identified through machine learning analysis of baseline QoL data, could serve as both prognostic markers and, more importantly, predictive markers for differential treatment benefit from atezolizumab in patients with aNSCLC. Specifically, we sought to develop and validate a QoL-based stratification model using data from four randomized clinical trials (IMpower150, BIRCH, OAK, POPLAR) to offer a complementary and accessible approach to traditional molecular biomarkers for guiding atezolizumab therapy in aNSCLC.

## 2 Materials and methods

### 2.1 Study design

**Figure 1:**
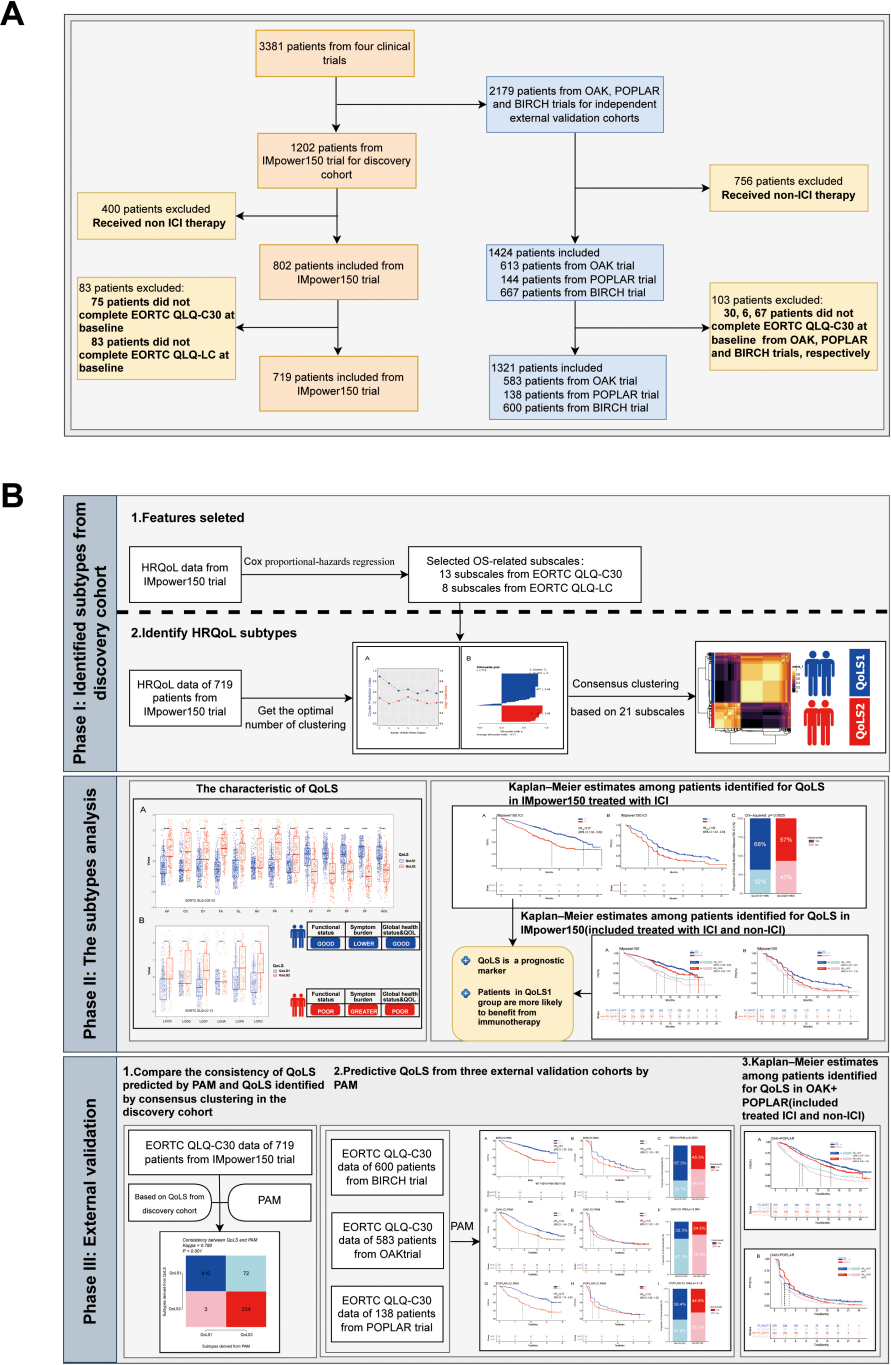
The flowchart of the study. The flowchart of patients inclusion and exclusion criteria **(A)**. Framework of the study with three phases **(B)**. ICI, immune checkpoint inhibitors. QoLS, Quality of life subtypes. EORTC QLQ-C30, European Organization for Research and Treatment of Cancer Quality of Life Questionnaire Core30; EORTC QLQ-C30, European Organization for Research and Treatment of Cancer Lung Cancer 13; The cohorts are from four international, multicenter studies (IMpower150, OAK, POPLAR and BIRCH).

The study flowchart is displayed in **Figure 1**. Phase I: Identified Quality of Life Subtypes(QoLS) by consensus clustering from discovery cohort. Phase II: Analyzed the association between QoLS and clinical outcomes. Phase III: Validated in three external validation cohorts. We followed the reporting guideline for the Transparent Reporting of a Multivariable Prediction Model for Individual Prognosis or Diagnosis (TRIPOD) (24).

### 2.2 Data source and patients

We enrolled four clinical trials of aNSCLC patients received atezolizumab: IMpower150(NCT02366143) (25), BIRCH (NCT02031458) (26), POPLAR (NCT01903993) (27), and OAK (NCT02008227) trials (28), provided through vivli.org. All trials’ protocols and CONSORT flowcharts have been previously published (25–28). Patients who received atezolizumab(ICI) were analyzed regardless of whether they had received platinum-based chemotherapy prior to that period. ICI-treated patients in the Phase III IMpower150 trial were used as the discovery cohort, In contrast, ICI-treated patients in the Phase II BIRCH, POPLAR and Phase III OAK trials were used as the external validation cohorts. Baseline QoL was measured in these clinical trials utilizing the EORTC QLQ-C30 and EORTC QLQ-LC questionnaires. For the discovery cohort, Patients who completed both the QLQ-C30 and the QLQ-LC questionnaires in full at baseline were included. The external validation cohorts included patients who completed the QLQ-C30 questionnaires in full at baseline. To assure the integrity and consistency of the data, improve the reliability of the cluster analysis and the interpretability of the results. Patients with incomplete or missing questionnaire data were excluded from the analysis. We also analyzed patients who received only chemotherapy (non-ICI) from the control arms of IMpower150, OAK, and POPLAR. Our study which included ethics section was approved by Vivli’s independent review panel.

### 2.3 Quality-of-life scales

QoL in lung cancer patients was assessed using the EORTC QLQ-C30 version 3 (29) and EORTC QLQ-LC version 13 (30). QLQ-C30 is a widely utilized patient-reported outcome (PRO) questionnaire for assessing functional status, Global Health Status, and symptom burden in cancer patients (31). The QLQ-C30 contains 30 items that are organized into six single-item scales (Appetite Loss, Dyspnea, Insomnia, Diarrhea, Constipation, Financial Difficulties), five functional scales (Physical, Role, Social, Emotional, Cognitive), three symptom scales (Fatigue, Pain, Nausea/Vomiting), and a Global Health Status/(Qol)QL scale (29). EORTC QLQ-LC13, regarded one of the standard tools for assessing QoL in patients with lung cancer, is a modular supplement to the QLQ-C30 (32). The module contains 13 items assessing lung cancer-related symptoms(coughing, hemoptysis, dyspnea, pain in chest, pain in arm or shoulder and pain elsewhere), as well as treatment-related adverse events (sore mouth, dysphagia, peripheral neuropathy and alopecia) (30). For all scales, item scores are summed and linearly converted to a scale of 0 to 100. Better health status is reflected by higher scores on the functional and Global Health Status scales, whereas greater symptom burden is indicated by higher scores on the symptom scales (33).

### 2.4 Clinical outcomes

The primary outcome was Overall survival(OS). OS was defined as the time period from the date of randomization to the date of death from any cause. The secondary outcomes were Progression free survival (PFS) and clinical benefit(CB). PFS was defined as the difference in time between the time between the date of randomization grouping and the date of first recorded Progressive Disease (PD) or death, whichever occurred earlier. CB was defined as complete remission (CR), partial remission (PR), or stable disease (SD) without progression for at least 6 months following the first ICI infusion (SD ≥6 months), as established in previous studies (34). The definitions of CR, PR, SD, and PD followed the RECIST v1.1 criteria (35).

### 2.5 Feature selection and preparation of the cohorts

We aimed to develop a quality-of-life subtype (QoLS) classification system using baseline QoL data for ICI-treated aNSCLC. To enhance comparability, we standardized the numerical data from both QLQ-C30 and QLQ-LC scales. Using univariate Cox proportional hazards regression, we extracted factors most relevant to OS, selecting those with a *p* ≤ 0.05, resulting in inclusion of 13 items from QLQ-C30 and 8 items from QLQ-LC (**Supplementary Table S1**). We calculated the Clustering Prediction Index (CPI), gap statistics and Silhouette score to determine the optimal number for clustering.

### 2.6 Clustering approaches based on baseline QoL data from ICI-treated aNSCLC

First, we explored the clustering performance of individual clustering algorithms on QoL data from each cohort. We applied 11 unsupervised methods (36) to identify potential subtypes with code availability being an important criterion for method selection(**Supplementary Table S2**). The performance of these individual clustering methods was evaluated using both internal clustering validation measures(Silhouette Coefficient (*SC*) (37), Davies-Bouldin Score (*DB*), and Calinski-Harabasz(CH) indices) (38, 39), and an assessment of their capacity to differentiate patient outcomes (40). Specifically, for each clustering result, we examined the association between the identified subtypes and key clinical outcomes, including OS, PPFS, and CB. This initial evaluation indicated that a more robust approach was needed to consistently identify clinically relevant subtypes across cohorts. To identify more robust and reproducible clustering subtypes, we then employed a consensus clustering approach integrating ten different clustering algorithms available in the MOVICS (V.0.99.17) package (41), concluding iClusterBayes, moCluster, CIMLR, IntNMF, PINSPlus, SNF, and LRA, ConsensusClustering, COCA, NEMO. Consensus clustering was performed on the baseline QoL data from the IMpower150 discovery cohort. To determine the optimal number of clusters (k), we utilized several criteria: the Clustering Prediction Index (CPI), Gap statistics, and visualization of the consensus matrix. Furthermore, the selection of the optimal number of clusters was critically guided by the ability of the resulting subtypes to stratify patients based on the aforementioned key clinical outcomes (OS, PFS, and CB) in the discovery cohort. The quality and separation of the final chosen clusters were further assessed using silhouette analysis. The final identified QoLS were considered robust and clinically meaningful based on the combined evaluation from statistical clustering indices, the stability of the consensus matrix, and their demonstrated capacity to differentiate patient prognosis and treatment response according to the criteria described above.

### 2.7 Prediction of QoLS using PAM based on QLQ-C30 data from ICI-treated aNSCLC

To enhance the applicability of the QoLS identified in the discovery cohort to other cohorts and real-world settings, we used partition around medoids(PAM) classifier to predict subtypes in the validation cohort based solely on QLQ-C30 data, given that adding QLQ-LC data increases model complexity and potential data loss without improving outcomes. First, PAM classifier was trained in the discovery cohort to predict the subtypes of the patients in the external validation cohorts, respectively, and each patient in the validation cohorts was matched a subtype label with the centroid that has the highest Pearson correlation with the patient. Secondly, the similarity and reproducibility of the subtypes obtained between the discovery and validation cohorts was assessed using inter-group proportion (IGP) statistics (42). Finally, we used Kappa statistics to compare the consistency of QoLS with the predicted subtypes by PAM. The clinical relevance of the predicted subtypes in the external cohorts was subsequently validated by examining their association with clinical outcomes.

### 2.8 Statistical analysis

The IMpower150, BIRCH, OAK and POPLAR trials were conducted from Match 2015 to September 2019, January 2012 to May 2015, Match 2014 to July 2016 and August 2013 to November 2015, respectively. This secondary analysis was conducted between November 2023 to May 2024. Continuous variables are shown as mean ± standard deviation (SD) or median (interquartile range, IQR). Categorical variables were reported as frequencies and percentages. The Kaplan-Meier method estimated median OS and PFS, which were compared between two QoLS groups using a stratified log-rank test at the two-sided significance level. Multivariate Cox proportional hazards regression models included baseline variables that were considered clinically relevant or that showed a univariate relationship with outcome. Variables were carefully selected for inclusion to ensure parsimony of the final models, given the number of events available. Multivariate analysis included QoLS, EOCG PS, and baseline PD-L1. All tests were two-tailed; *p* ≤ 0.05 was considered to be statistically significant. The R in Vivli platform (V.4.2.2, R Core Team 2022) was used for all statistical analyses.

## 3 Results

### 3.1 Patient baseline characteristics

This study included 2040 aNSCLC patients received atezolizumab from four global clinical trials. Among these, 719 patients in the discovery cohort received first-line atezolizumab combination therapy, 600 patients who were all positive for PD-L1 expression in the BIRCH study received atezolizumab monotherapy as first-line or subsequent therapy, 583 and 138 patients who had failed Platinum-Containing therapy received atezolizumab monotherapy as second- or third-line treatment in the OAK and POPLAR study, respectively. The clinicopathological characteristics are listed in **Supplementary Table S3**. A higher proportion of patients in the validation cohorts had an ECOG PS of 1 and more than three metastatic sites compared to the discovery cohort. Except for the discovery cohort (all patients with non-squamous NSCLC), the other three external validation cohorts included approximately 2/3 patients with non-squamous NSCLC.

### 3.2 Clustering performance of single clustering algorithms

To evaluate the clustering performance of individual clustering algorithms on QoL data, we applied 11 clustering methods to identify different subtypes from QoL data treated with atezolizumab in IMpower150(719 patients), OAK (551patients), BIRCH (555 patients) and POPLAR(126 patients) trials. We found that the clustering performance metrics demonstrate that each clustering method is less effective when applied to QoL data from the four cohorts under consideration (**Supplementary Table S4**). Furthermore, we observed a significant difference in OS and PFS between clust1 and clust2 groups identified by the majority of methods in IMpower150 trial. However, almost all clustering methods employed in OAK, POPLAR and BIRCH trials did not demonstrate a statistically significant differentiation for OS, PFS and CB between two subtypes (**Supplementary Figures S1**-**S4**).

### 3.3 Identification of QoLS in the discovery cohort by consensus clustering

**Figure 2:**
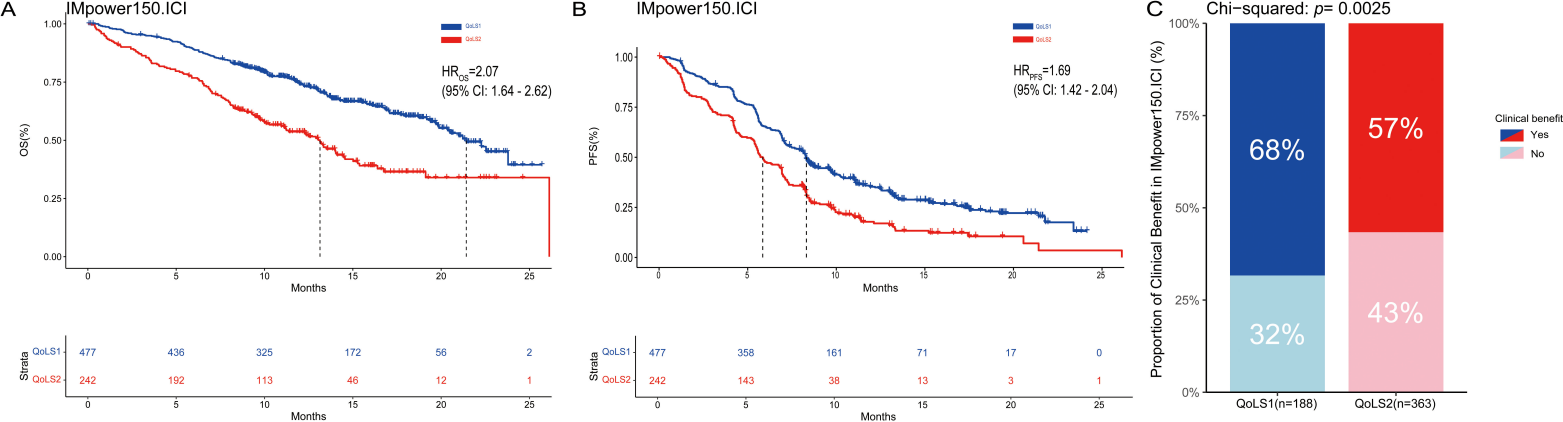
Kaplan-Meier Survival Curves and rate of clinical benefit for identified QoLS Group via consensus clustering in discovery dataset. **(A)** Kaplan-Meier plots for OS of the two identified QoLSs. **(B)** Kaplan-Meier plots for PFS of the two identified QoLSs. **(C)** Bar charts for clinical benefit rates of the two identified QoLSs. QoLS, Quality of life subtypes. OS, Overall survival. PFS, Progression free survival.

In order to identify more robust and reproducible clustering subtypes, ten clustering algorithms were used from the MOVICS (V.0.99.17) package, which were then integrated into a robust classification via consensus clustering. Based on the recommended number of clusters from the Clustering Prediction Index (CPI) and Gap statistics, we selected two clusters to identify the Quality of Life Subtypes(QoLS) (**Supplementary Figures S5A**, **S6A**). The results of the silhouette analysis demonstrated the general similarity of the patients in each cluster, with silhouette values of 0.64 and 0.80 for QoLS1 and QoLS2 respectively (**Supplementary Figure S6B**). We identified two distinct quality of life (QOL) subtypes among the patient cohort: QoLS1 (n=477) and QoLS2 (n=242). Patients in the QoLS1 group were predominantly characterized by better functional status and health status, lower symptom burden and financial stress, while the opposite was true for patients in the QoLS2 group (**Supplementary Figures S5B**, **S7A, SB**). We observed a significant differences existed in racial distribution (QoLS1 had a higher Asian proportion *vs*.QoLS2; p=0.003), ECOG PS (more QoLS1 with ECOG PS 0, fewer with ECOG PS ≥1; p<0.001), and metastasis number (more QoLS2 with >3 metastases; p<0.001), all detailed in **Table 1**. Importantly, the identified QOL subtypes demonstrated significant prognostic value for clinical outcomes. QoLS2 group had shorter median OS (13.14 *vs*. 21.42 months, hazard ratio (HR)2.07, 95%CI 1.64 to 2.62; *p <*0.0001, **Figure 2A**) and PFS (5.7 *vs*. 8.3 months, HR 1.69, 95%CI 1.42 to 2.04; *p* < 0.0001, **Figure 2B**) compared to QolS1. Clinical benefit rates for QoLS2 were 57% compared to 68% for QoLS1(*p*=0.0027, **Figure 2C**). Time-dependent AUC predicting OS and PFS at 6, 12 and 24 months were 0.61, 0.6 and 0.53, respectively, as shown in **Supplementary Figure S8**.

**Table 1:** Differences in characteristics between QOLS1 and QOLS2 in the discovery cohort.

Variables	Total(719)	QoLS1(477)	QoLS2(242)	*p*
AGE, Median (Q1,Q3)	63 (57, 69)	64 (57, 69)	63 (56, 69)	0.462
SEX, n (%)				0.076
Female	278 (38.7)	173 (36.3)	105 (43.4)	
Male	441 (61.3)	304 (63.7)	137 (56.6)	
RACE, n (%)				0.003
Asian	101 (14)	79 (16.6)	22 (9.1)	
White	577 (80.3)	379 (79.5)	198 (81.8)	
Other	19 (2.6)	8 (1.7)	11 (4.5)	
Missing	22 (3.1)	11 (2.3)	11 (4.5)	
ECOG, n (%)				< 0.001
0	299 (41.6)	232 (48.6)	67 (27.7)	
≥1	417 (58)	242 (50.7)	175 (72.3)	
Unknown	3 (0.4)	3 (0.6)	0 (0)	
Smoking history, n (%)				0.166
Current	167 (23.2)	106 (22.2)	61 (25.2)	
Never	147 (20.4)	107 (22.4)	40 (16.5)	
Previous	405 (56.3)	264 (55.3)	141 (58.3)	
Liver metastasis, n (%)				0.075
Absent	627 (87.2)	424 (88.9)	203 (83.9)	
Present	92 (12.8)	53 (11.1)	39 (16.1)	
PD-L1, n (%)				0.37
Negative	342 (47.6)	230 (48.2)	112 (46.3)	
Positive	376 (52.3)	247 (51.8)	129 (53.3)	
Missing	1 (0.1)	0 (0)	1 (0.4)	
Number of metastasis, n (%)				< 0.001
≤3	695 (96.7)	470 (98.5)	225 (93)	
>3	24 (3.3)	7 (1.5)	17 (7)	

QoLS, Quality of life subtypes; ECOG PS, Eastern Cooperative Oncology Performance score; PD-L1, Programmed death-ligand 1; TC, Tumor cell; IC, Immune cell.

a PD-L1 positive TC1/2/3 and IC1/2/3; b PD-L1 negative TC0 and IC0.

P-values meeting the significance threshold (p < 0.05) are bolded for emphasis..

### 3.4 Prediction and validation of QoLS in ICI-treated external cohorts

**Figure 3:**
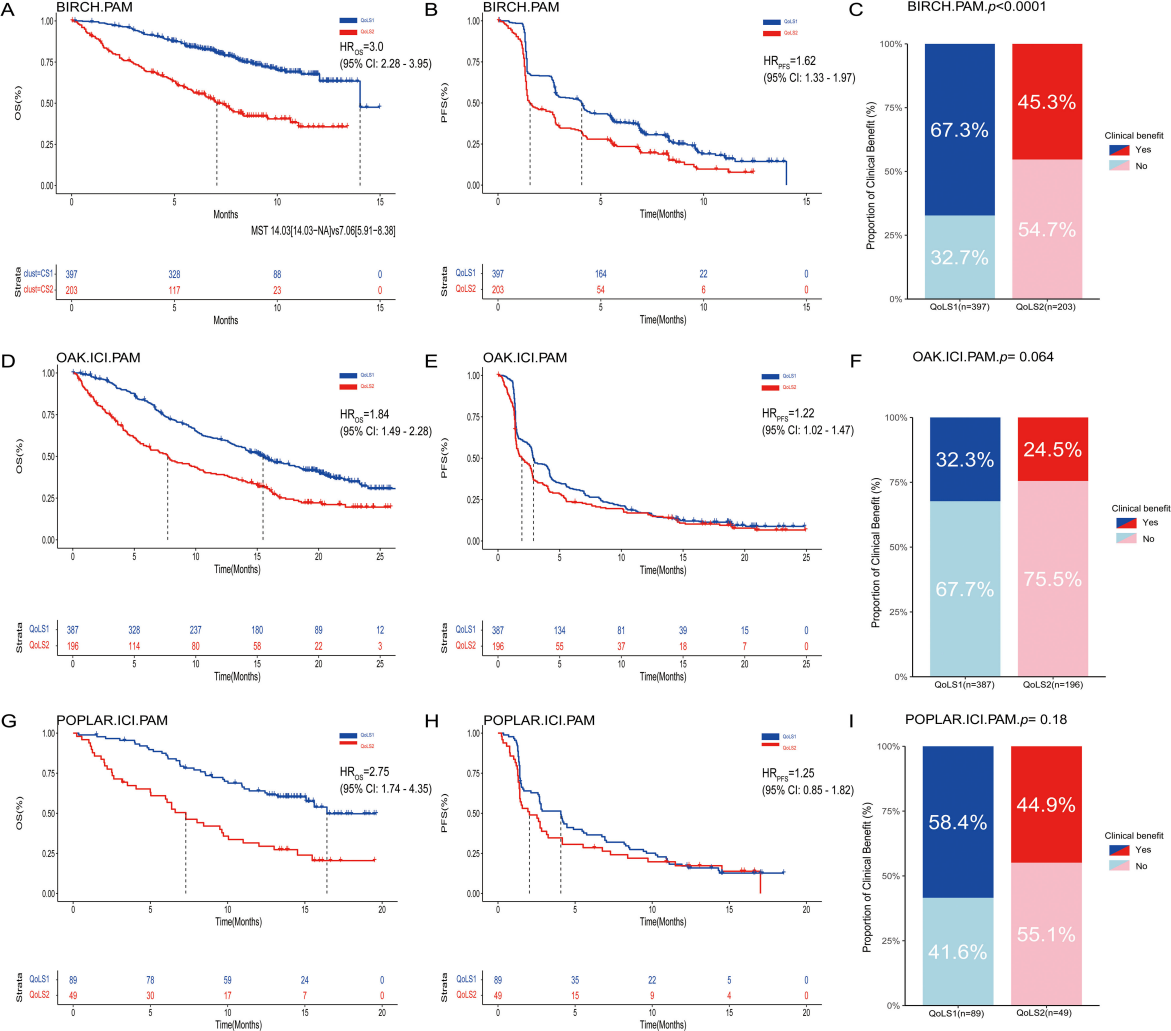
Kaplan-Meier Survival Curves and rate of clinical benefit for identified QoLS Group via consensus clustering in three validation datasets. Kaplan-Meier curve of OS **(A)**, PFS **(B)** and Bar charts for clinical benefit rates **(C)** of the two predicted QoLSs by PAM in BIRCH. Kaplan-Meier curve of OS **(D)**, PFS **(E)** and Bar charts for clinical benefit rates **(F)** of the two predicted QoLSs by PAM in OAK(treated with ICI) . Kaplan-Meier curve of OS **(G)**, PFS **(H)** and Bar charts for clinical benefit rates **(I)** of the two predicted QoLSs by PAM in POPLAR(treated with ICI). Analyses were conducted using LogRank tests and Chisq test. QoLS, Quality of life subtypes. PAM, partition around medoids. OS, Overall survival. PFS, Progression free survival.

In three external validation cohorts, patients were classified into two subtypes: 387 and 196 in OAK (IGP values 0.85, 0.78), 397 and 203 in BIRCH (IGP values 0.89, 0.74), and 89 and 49 in POPLAR (IGP values 0.89, 0.74) for CS1 and CS2, respectively. For discovery cohort, the Kappa value was 0.78 (*p* < 0.001), indicating a high level of consistency between predicted subtypes by PAM and QoLS (**Supplementary Figure S9**). Furthermore, we examined the distribution of key clinical characteristics between QoLS1 and QoLS2 within each external validation cohort. Similar to the discovery cohort, QoLS1 patients in the external cohorts generally exhibited better ECOG PS and a lower burden of metastases compared to QoLS2 patients. Differences in racial distribution between the subtypes were also assessed across these cohorts. These clinical characteristics analyses confirmed that the underlying patient profiles associated with each QOL subtype were largely consistent across the discovery and external validation datasets (**Supplementary Tables S5**, **S6**). The similarity of distribution of QoL data (**Supplementary Figure S10**) and survival outcomes can also be validated in external cohorts. QoLS2 was consistently associated with poor OS (BIRCH, HR 3.0, 95%CI 2.28 to 3.95, *p*<0.0001; OAK, HR 1.84, 95%CI 1.49 to 2.28, *p*<0.0001; POPLAR, HR 2.75, 95%CI 1.74 to 4.35, *p*<0.0001, **Figures 3A, D, G**) and PFS (BIRCH, HR 1.62, 95%CI 1.33 to 1.97, *p*< 0.0001; OAK, HR 1.22, 95%CI 1.02 to 1.47, *p*= 0.032; POPLAR, HR 1.25, 95%CI 0.85 to 1.82, *p*=0.257, **Figures 3B, E, H**) compared to QoLS1, showing consistent clinical outcomes across multiple validation cohorts, suggesting that patients from QoLS2 group achieved a significantly worse OS and PFS, except in the POPLAR cohort for PFS. In validation cohorts but BIRCH, we found no significant difference in CB between QoLS1 and QoLS2 (**Figures 3C, F, I**).

### 3.5 Cox regression and subgroup analysis

Additionally, we analyzed whether QoLS was an independently marker in univariate and multivariate Cox regression analyses of discovery and validation cohorts. In discovery and three validation cohorts, The QoLS2 had statistically significant shorter OS and PFS compared with those in the QoLS1 as demonstrated in univariate Cox regression analyses. We further performed multivariate Cox regression survival analyses to adjust for other clinical variables such as sex, age, smoking history, ECOG-PS, race, PD-L1 and number of metastatic, as only data sets with almost complete demographic information were assessed. Notably, multivariate survival analyses indicated that the QoLS remained to be an independent marker of survival outcomes after the adjustment (**Table 2**; **Supplementary Tables S7–S9**). Our subgroup analysis revealed that the association between QoLS and clinical outcomes was independent of sex, age, ECOG-PS, and PD-L1 expression(**Supplementary Figure S11**). We found that QoLS2 was more unfavorable than QoLS1 in HRs, whereas there was no significant difference between QoLS1 and QoLS2 in Asians, patients with more than 3 metastases at a distance (**Supplementary Figure S12**).

**Table 2:** Univariable and multivariable Cox proportional hazards analysis for OS and PFS in discovery cohort.

Variable	OS				PFS			
	Univariate HR(95%CI)	*p*	Multivariate HR(95%CI)	*p*	Univariate HR(95%CI)	*p*	Multivariate HR(95%CI)	*p*
Age group								
<65	Reference		Reference		Reference		Reference	
≥65	1.1 (0.87,1.38)	0.434	1.18 (0.93,1.5)	0.1659	0.97 (0.81,1.15)	0.6926	1.02 (0.85,1.22)	0.8295
Treatment								
ACP	Reference		Reference		Reference		Reference	
ABCP	0.86 (0.68,1.08)	0.1979	0.83 (0.66,1.04)	0.1116	0.69 (0.58,0.81)	**<0.0001**	0.64 (0.54,0.76)	**<0.0001**
oLS								
QoLS1	Reference		Reference		Reference		Reference	
QoLS2	2.05 (1.63,2.59)	**<0.0001**	1.72 (1.35,2.2)	**<0.0001**	1.69 (1.41,2.02)	**<0.0001**	1.66 (1.38,2.01)	**<0.0001**
ECOG PS								
0	Reference		Reference		Reference		Reference	
≥1	2.25 (1.74,2.9)	**<0.0001**	1.93 (1.48,2.5)	**<0.0001**	1.58 (1.32,1.89)	**<0.0001**	1.46 (1.22,1.76)	**<0.0001**
Number of metastatic								
≤3	Reference		Reference		Reference		Reference	
>3	3.09 (1.89,5.06)	**<0.0001**	2.15 (1.29,3.58)	**0.0032**	1.68 (1.08,2.63)	0.0226	1.42 (0.89,2.25)	0.1384
PD-L1								
Positive	Reference		Reference		Reference		Reference	
Negative	1.57 (1.24,1.98)	**0.0001**	1.56 (1.24,1.97)	**0.0002**	1.58 (1.32,1.87)	**<0.0001**	1.58 (1.32,1.88)	**<0.0001**
Race								
White	Reference		Reference		Reference		Reference	
Asian	0.58 (0.4,0.84)	**0.0041**	0.6 (0.41,0.88)	**0.009**	1.08 (0.85,1.38)	0.5157	1.05 (0.82,1.35)	0.7069
Other	1.08 (0.53,2.17)	0.8383	0.94 (0.46,1.91)	0.8579	1.33 (0.8,2.23)	0.273	1.07 (0.64,1.82)	0.7893
Unknown	0.99 (0.51,1.92)	0.9686	0.69 (0.31,1.57)	0.3789	0.71 (0.42,1.18)	0.187	0.58 (0.32,1.07)	0.0806
Sex								
Female	Reference		Reference		Reference		Reference	
Male	1.18 (0.93,1.5)	0.1837	1.14 (0.88,1.47)	0.327	0.98 (0.82,1.17)	0.8063	1.06 (0.87,1.29)	0.5534
Smoking history								
Never	Reference		Reference		Reference		Reference	
Previous	1.01 (0.8,1.27)	0.9214	1.15 (0.81,1.63)	0.4296	0.85 (0.71,1.01)	0.0578	0.78 (0.62,1)	0.0457
Current	1.32 (1.02,1.72)	**0.0382**	1.35 (0.91,2)	0.1412	1.08 (0.88,1.32)	0.4829	0.84 (0.63,1.11)	0.2169
Unknown	1.87 (0.6,5.84)	0.2814	-	-	1.2 (0.45,3.21)	0.7168	-	-

APC, Atezolizumab+Paclitaxel+Carboplatin; ABPC, Atezolizumab+Bevacizumab+Paclitaxel + Carboplatin; QoLS, Quality of life subtypes; ECOG PS, Eastern Cooperative Oncology Performance score; PD-L1, Programmed death-ligand 1; OS, Overall survival; PFS, Progression free survival; TC, Tumor cell; IC, Immune cell.

a PD-L1 positive TC1/2/3 and IC1/2/3; b PD-L1 negative TC0 and IC0.

The bold values indicate statistically significant results (p < 0.05).

### 3.6 Predictive role of QoLS in clinical outcomes for ICI-treat aNSCLC

**Figure 4:**
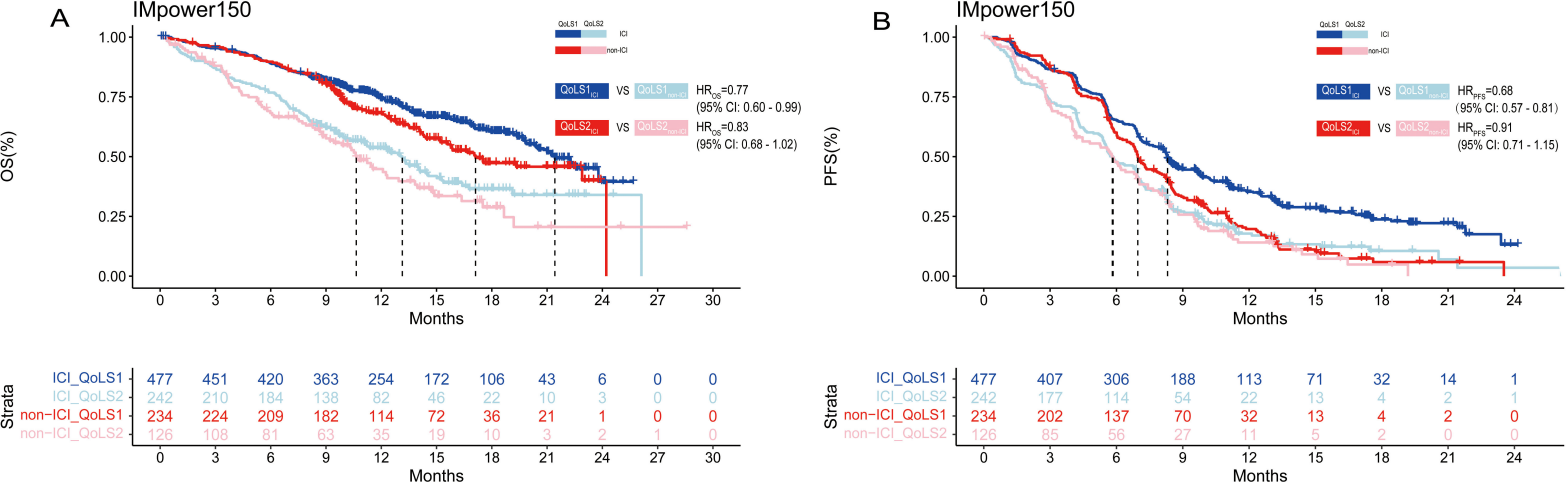
Kaplan–Meier estimates among patients identified for QoLS in IMpower150(included treated ICI and non-ICI). Kaplan–Meier estimates of OS **(A)** in patients evaluated for QoLS1 treated with ICI (dark blue) and QoLS1 patients treated with non ICI (dark red), and comparing QoLS2 patients treated with treated with ICI (light blue) and QoLS2 patients treated with non ICI (light red). Kaplan–Meier estimates of PFS **(B)** in patients evaluated for QoLS1 treated with ICI (dark blue) and QoLS1 patients treated with non ICI (dark red), and comparing QoLS2 patients treated with treated withICI (light blue) and QoLS2 patients treated with non ICI (light red). OS, Overall survival. PFS, Progression free survival. ICI, immune checkpoint inhibitor.

We also performed survival analyses of the OAK, POPLAR and IMpower150 trials to test the therapeutic predictive function of QoLS. Due to the similarities in the design of the OAK and POPLAR trials, the data from these two trials were pooled together. Our developed QoLS is a robust predictive model for OS in ICI-treated aNSCLC patients, as compared to non-ICI-treated patients, in the IMpower150(QoLS1: HR _ICI_ *vs*. _non-ICI_ = 0.77, 95%CI 0.60 to 0.99, *p* = 0.04; QoLS2: HR _ICI_ *vs*. _non-ICI_ = 0.83, 95%CI 0.62 to 1.10, *p* = 0.2) (**Figure 4A**) and pooled OAK and POPLAR cohorts(QoLS1: HR _ICI_ *vs*. _non-ICI_ = 0.79, 95%CI 0.67 to 0.93, *p* = 0.007; QoLS2: HR _ICI_ *vs*. _non-ICI_ = 0.83, 95%CI 0.68 to 1.02), *p* = 0.07) (**Supplementary Figure S13A**). PFS was statistically significant between the ICI and non-ICI treatment groups for QoLS1 in the IMpower150 trial(QoLS1: HR _ICI_ *vs*. _non-ICI_ =0.68, 95%CI: 0.57 to 0.81, *p* < 0.0001; QoLS2: HR _ICI_ *vs*. _non-ICI_ =0.91, 95%CI: 0.71 to 1.15, *p* = 0.4) (**Figure 4B**), while no significant difference in the pooled OAK and POPLAR studies (QoLS1: HR _ICI_ *vs*. _non-ICI_ = 0.97, *p* = 0.7; QoLS1: HR _ICI_ *vs*. _non-ICI_ = 0.95, *p* = 0.6) (**Supplementary Figure S13B**), in consistence to the findings of the two studies (27, 28), strengthening the fact that QoLS we developed is a predictive and prognostic marker, especially for OS.

In summary, these findings suggest that QoLS could predict the OS of aNSCLC patients received atezolizumab to determine whether treatment can be discontinued, or consider alternative treatment plans. There was no significant difference in clinical benefit between ICI and non-ICI therapy across the QoLS1 and QoLS2 groups (**Supplementary Figure S14**).

## 4 Discussion

In this study, we successfully applied an integrative machine learning approach, specifically consensus clustering, to analyze baseline QoL data from a large cohort of patients with aNSCLC receiving atezolizumab. This analysis allowed us to identify two distinct and clinically relevant QoLS, designated QoLS1 and QoLS2. Our findings demonstrate that these QoLS serve as robust prognostic markers for survival outcomes and, importantly, possess predictive value for differential treatment benefit from atezolizumab in aNSCLC patients, findings consistently validated across multiple independent external clinical trial cohorts.

The identified QoLS exhibited clear differences in baseline QoL profiles. Patients in the QoLS1 group were characterized by a better overall QoL, encompassing superior functional status (physical, role, social, emotional), better global health status, and lower symptom burden (fatigue, pain, nausea/vomiting, etc.) and financial difficulties. Conversely, the QoLS2 group presented with a poorer QoL profile across these domains. These distinct QoL profiles likely reflect underlying differences in patients’ overall health status, disease burden, psychological well-being, and resilience. The observed association between better baseline QoL (QoLS1) and better prognosis (OS, PFS) aligns with numerous previous studies demonstrating the prognostic value of PROs and QoL in various cancers, including NSCLC (17, 19, 20, 43, 44). For instance, poorer physical performance (45), emotional distress (18), higher symptom burden (46), and financial difficulties (44) have all been linked to worse outcomes. Social functioning and support networks may influence patients’ treatment adherence and rehabilitation outcomes (47). Our findings consolidate these individual QoL aspects into distinct patient subtypes, providing a more holistic view of how baseline patient-reported status relates to prognosis.

Beyond prognosis, a key contribution of our study is the demonstration that these QoLS can predict differential benefit from atezolizumab. Patients in the QoLS1 group derived a significant survival advantage from atezolizumab compared to non-ICI treatments, suggesting they are a population particularly likely to benefit from this therapy. In contrast, patients in the QoLS2 group did not show a statistically significant differential survival benefit from atezolizumab over non-ICI treatments. This predictive capacity, particularly for Overall Survival(OS), is highly relevant in the clinical management of aNSCLC. OS is widely regarded as the most definitive and clinically meaningful endpoint in advanced cancer therapy, as it directly measures the impact of treatment on prolonging a patient’s life. Unlike progression-free survival (PFS), which assesses the duration of disease control, OS is less susceptible to confounding factors like subsequent therapies and more directly reflects the ultimate benefit of a treatment regimen. Our finding that QoLS robustly predicts OS across multiple independent validation cohorts is therefore of significant potential clinical utility for potentially informing these critical management decisions. While our analysis in the pooled OAK/POPLAR cohorts did not show a statistically significant predictive effect for progression-free survival (PFS) benefit, the consistent prediction of the more clinically definitive endpoint, OS, further underscores the primary strength and potential clinical relevance of our QoLS marker in guiding treatment strategy. While previous studies have linked better performance status (often correlated with QoL) to better ICI response (45), our study, using a comprehensive QoL assessment and machine learning, identifies specific subtypes with differential treatment responses.

While our study focused on the clinical utility of QoLS derived from patient-reported outcomes, and did not involve direct investigation into the underlying molecular mechanisms, the existing literature provides potential biological explanations for the observed differential responses to immunotherapy among these subtypes. The symptoms that define these QoLS, such as chronic pain, fatigue, and emotional distress, are known to be associated with physiological changes that can modulate the immune system and potentially impact the efficacy of ICIs. Specifically, chronic symptom burden can activate neuroendocrine pathways and induce systemic inflammatory responses. Activation of the sympathetic nervous system (SNS), a key component of the stress response, has been shown to inhibit T cell responses (48). Furthermore, stress-induced immunosuppression may involve processes like macrophage pyroptosis (49). Concurrently, systemic inflammation is often linked to clusters of neuropsychological symptoms (50). Clinical studies support these potential links. For instance, patients experiencing pain, fatigue, depression, and sleep disorders have been found to exhibit poorer performance status and significantly higher levels of interleukin-6 (IL-6) (46). Elevated IL-6 is a critical mediator that can promote various immunosuppressive mechanisms, including enhancing the activity of myeloid-derived suppressor cells (MDSCs), which are known to contribute to ICI resistance (51). A prospective study involving 227 patients with advanced NSCLC showed that baseline emotional distress (ED) was associated with shorter progression-free survival and a lower objective response rate after ICI treatment. The mechanism may be related to elevated cortisol levels (18), a finding that aligns with preclinical models demonstrating stress-induced, glucocorticoid-dependent T cell apoptosis. The mechanisms linking baseline QoL to differential ICI efficacy are complex and multifactorial. Further research is needed to elucidate these potential biological underpinnings.

### 4.1 Strengths and limitations

A major strength of this study is the use of a large dataset derived from four global, randomized clinical trials, providing a robust foundation for the analysis. The external validation in three independent cohorts significantly enhances the reliability and generalizability of our findings regarding the prognostic value of QoLS. Unlike prior work focusing on individual QoL items or clustering of non-QoL data (19, 23, 52, 53), we used consensus clustering on baseline QoL to define QoLS. This study is the first to show these QoLS are both prognostic and predictive for atezolizumab differential benefit in aNSCLC, validated across multiple cohorts. The non-invasive nature and accessibility of QoL assessment are also key advantages.

However, our study has several limitations. First, the discovery and validation cohorts were limited to patients receiving atezolizumab. While our findings are robust for this specific agent, it remains unclear whether similar QoLS can be identified or hold the same predictive value in patients treated with other ICIs (e.g., nivolumab, pembrolizumab) or combination immunotherapies. Future research is needed to validate QoLS across different ICI regimens. Second, while the QLQ-C30 was used for prediction in validation cohorts for broader applicability, the exclusion of lung cancer-specific symptoms from the QLQ-LC in this prediction step might limit the capture of disease-specific nuances. Although combining QLQ-C30 and QLQ-LC did not significantly improve overall model performance in the discovery phase, the specific contribution of LC-related symptoms to prediction warrants further investigation. Third, while the overall cohort size is large, the sample size within certain subgroups (e.g., Asian patients, patients with more than three metastatic sites) was relatively limited. This may affect the robustness and generalizability of subgroup analysis findings and highlights the need for validation in larger, more diverse populations to confirm the universal applicability of QoLS. Finally, our study is based on clinical trial data and does not include molecular analyses to explore the biological mechanisms underlying the association between QoLS and differential ICI response.

Based on our findings and limitations, several future research directions are warranted. Prospective studies are needed to validate the clinical utility of QoLS in guiding treatment decisions. Further research should explore the predictive value of QoLS in patients treated with a broader range of ICI agents and combinations. Investigating the potential added value of incorporating lung cancer-specific QoL domains (from QLQ-LC) into predictive models is also important. Future studies should aim to validate QoLS in larger and more diverse real-world patient populations to ensure generalizability. Finally, exploring the biological basis of the identified QoLS and their association with immune response and treatment outcomes could provide deeper mechanistic insights and pave the way for integrating QoL data with molecular biomarkers to develop more precise and personalized predictive models.

## 5 Conclusion

In conclusion, our study demonstrates the significant potential of leveraging integrative machine learning to analyze patient-reported QoL data for identifying prognostic and predictive subtypes in aNSCLC patients undergoing atezolizumab immunotherapy. This approach offers a valuable, non-invasive tool that could enhance personalized treatment decision-making and improve outcomes in this patient population.

## Data Availability

The datasets presented in this article are not readily available due to patient privacy. Qualified researchers may request access to individual patient-level data through the clinical study data request platform (Vivli, Inc., https://vivli.org/). For further details please refer to Roche’s Global Policy on the Sharing of Clinical Information and how to request access to related clinical study documents; see https://www.roche.com/innovation/process/clinical-trials/data-sharing/ and https://vivli.org/ourmember/roche/. Requests to access the datasets should be directed to https://www.roche.com/innovation/process/clinical-trials/data-sharing/ and https://vivli.org/ourmember/roche/.
