# Supplementary material for: Quality-of-life scale machine learning approach to predict immunotherapy response in patients with advanced non-small cell lung cancer
Source: Front Immunol. 2025 Jul 18;16:1600265. doi: 10.3389/fimmu.2025.1600265 (PMC12314532; doi:10.3389/fimmu.2025.1600265)
Supplement: Supplementary file 1 [file DataSheet1.docx]

Supplementary Material

# Supplementary Figures and Tables

## Supplementary Figures

**
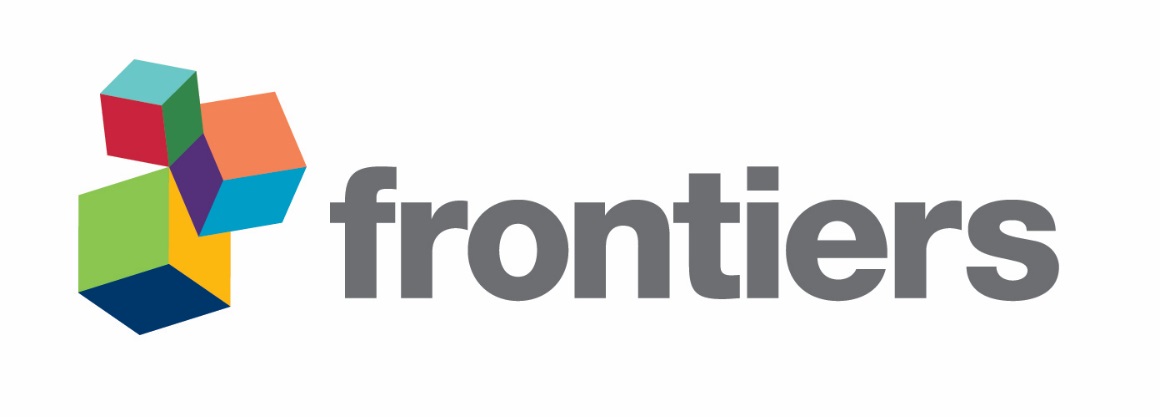
**

**Supplementary Figure 1.** **Kaplan-Meier Survival Curves and rate of clinical benefit of identified subtypes based on the QoL data by single clustering method on IMpower150 study(IC I treated).** (Panel A) Kaplan-Meier plots for OS of the two subtypes identified by 11 clustering algorithms ,respectively

Abbreviations: OS, Overall survival. PFS, Progression free survival. RGCCA, regularized Generalized Canonical Correlation Analysis. MCIA, Multiple Co-Inertia Analysis. NMF, Non-negative Matrix factorization. SNF, Similar Network Fusion. CIMLR, Cancer Integration via Multikernel Learning. QoLS, Quality of life subtypes. ICI, Immune Checkpoint Inhibitor

**Supplementary Figure 1.Continued.** (Panel B) Kaplan-Meier plots for PFS of the two subtypes identified by 11 clustering algorithms ,respectively

**Supplementary Figure 1.Continued.** (Panel C) Bar charts for clinical benefit rates of the two subtypes identified by 11 clustering algorithms ,respectively

**Supplementary Figure 2. Kaplan-Meier Survival Curves and rate of clinical benefit of identified subtypes based on the QoL data by single clustering method on OAK study(IC I treated)** (Panel A) Kaplan-Meier plots for OS of the two subtypes identified by 11 clustering algorithms ,respectively

**Supplementary Figure 2.Continued.** (Panel B) Kaplan-Meier plots for PFS of the two subtypes identified by 11 clustering algorithms ,respectively

**Supplementary Figure 2.Continued.** (Panel C) Bar charts for clinical benefit rates of the two subtypes identified by 11 clustering algorithms ,respectively

**Supplementary Figure 3. Kaplan-Meier Survival Curves and rate of clinical benefit of identified subtypes based on the QoL data by single clustering method on BIRCH study.** (Panel A) Kaplan-Meier plots for OS of the two subtypes identified by 11 clustering algorithms ,respectively

**Supplementary Figure 3.Continued.** (Panel B) Kaplan-Meier plots for PFS of the two subtypes identified by 11 clustering algorithms ,respectively

**Supplementary Figure 3.Continued.** (Panel C) Bar charts for clinical benefit rates of the two subtypes identified by 11 clustering algorithms ,respectively

**Supplementary Figure 4. Kaplan-Meier Survival Curves and rate of clinical benefit of identified subtypes based on the QoL data by single clustering method on POPLAR studies.** (Panel A) Kaplan-Meier plots for OS of the two subtypes identified by 11 clustering algorithms ,respectively

**Supplementary Figure 4.Continued.** (Panel B) Kaplan-Meier plots for PFS of the two subtypes identified by 11 clustering algorithms ,respectively

**Supplementary Figure 4.Continued.** (Panel C) Bar charts for clinical benefit rates of the two subtypes identified by 11 clustering algorithms ,respectively

**Supplementary Figure 5. QoLS visualization identified by consensus clustering on the discovery dataset.** (A) features of the QolS1 and QolS2 subtypes. (B) Clustering of Discovery set through 10 cutting-edge multi-omics clustering methods. QoLS, Quality of life subtypes.

Abbreviations: AP, Appetite Loss. CO, Constipation. DY, Dyspnoea. FA , Fatigue. SL, Insomnia. NV, Nausea/Vomiting. PA, Pain. FI, Financial difficulties. EF, Emotional Functioning. PF Physical Functioning. RF, Role Functioning. SF, Social Functioning. QOL, Quality of life. LCCO, coughing. LCHA, haemoptysis. LCDY, dyspnoea. LCPA, pain inarm or shoulder. LCPO, pain in other parts. LCDS, dysphagia. QoLS, Quality of life subtypes.

**Supplementary Figure 6. Identification of QoLS by Consensus Clustering in discovery dataset.** (A) Determination of the optimal number of clusters using the cluster prediction index and Gap-statistics. (B) Evaluation of sample homogeneity through silhouette scores derived from consensus ensemble result.

**Supplementary Figure 7. Distribution of patient QoL scale data (standardized) in discovery dataset.** Comparison of Quality of Life Subtypes (QoLS1 vs QoLS2) Across EORTC QLQ-C30 V3 (A) and QLQ-LC13(B) Domains

**Supplementary Figure 8. The performance of QoLS for predicting OS and PFS in discovery dataset.** (A) The performance of QoLS for predicting OS after administration with ICI was evaluated using time-dependent AUC values in discovery dataset. (B)The performance of QoLS for predicting PFS after administration with ICI was evaluated using time-dependent AUC values in discovery dataset.

Abbreviations: AUC, ‎Area under the curve; ROC, Receiver operating characteristic

**Supplementary Figure 9. Consistency analysis of identified QoLS by Consensus Clustering and predictived QoLS by PAM in discovery dataset.** QoLS Kappa value of IMpower150 (Kappa = 0.78, p < 0.001) showed high similarity between PAM and QoLS (QoLS1 vs. QoLS1 and QoLS2 vs. QoLS2).

**Supplementary Figure 10. Distribution of patient QoL scale data (standardized) in validation cohorts.** Comparison of QoLS(QoLS1 vs QoLS2) across EORTC QLQ-C30 V3 (A) and QLQ-LC13(B) domains in OAK study(treated with ICI)

**Supplementary Figure 10. Continue.** Comparison of QoLS(QoLS1 vs QoLS2) across EORTC QLQ-C30 V3 (C) and QLQ-LC13(D) domains in BIRCH study

**Supplementary Figure 10. Continue.** Comparison of QoLS(QoLS1 vs QoLS2) across EORTC QLQ-C30 V3 (E) and QLQ-LC13(F) domains in POPLAR study(treated with ICI)

**Supplementary Figure 11. Subgroup analyses in validation cohorts.** Effect of different clinical variables on OS(A), PFS(B) and clinical benefit(C) in OAK study(treated with ICI)

**Supplementary Figure 11. Continue.** Effect of different clinical variables on OS(D), PFS(E) and clinical benefit(F) in POPLAR study(treated with ICI)

**Supplementary Figure 11. Continue.** Effect of different clinical variables on OS(G), PFS(H) and clinical benefit(I) in BIRCH study

**Supplementary Figure 12. Forest Plot of OS and PFS by Subgroup in discovery cohort.** Effect of different clinical variables on OS(A), PFS(B)

Abbreviations:ECOG-PS, Eastern Cooperative Oncology Group Performance Status. PD-L1, Programmed Cell Death Ligand 1. ACP, Atezolizumab+Paclitaxel+Carboplatin. ABCP, Atezolizumab+Bevacizumab+Paclitaxel + Carboplatin. QoLS, Quality of life subtypes. OS, Overall survival. PFS, Progression free survival.

**Supplementary Figure 13. Kaplan–Meier estimates among patients predicted for QoLS in the pooled OAK and POPLAR study(included treated ICI and non ICI).** Kaplan–Meier estimates of OS (A) in patients evaluated for QoLS1 treated with ICI (dark blue) and QoLS1 patients treated with non ICI (dark red) , and comparing QoLS2 patients treated with treated withICI (light blue) and QoLS2 patients treated with non ICI (light red). Kaplan–Meier estimates of PFS (B) in patients evaluated for QoLS1 treated with ICI (dark blue) and QoLS1 patients treated with non ICI (dark red), and comparing QoLS2 patients treated with treated withICI (light blue) and QoLS2 patients treated with non ICI (light red) .


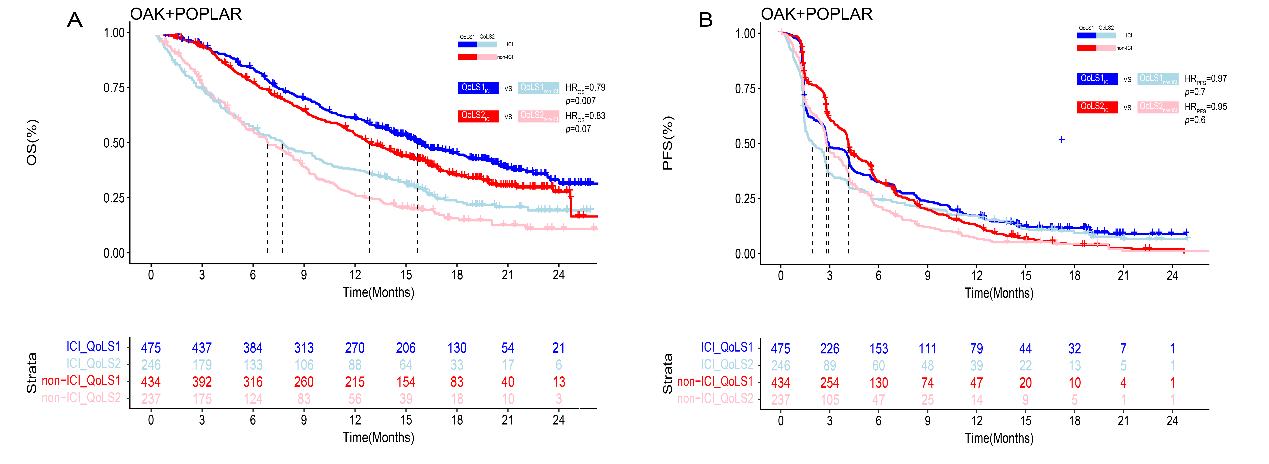


**Supplementary Figure 14. Rate of clinical benefit for QoLS Group in IMpower150 and the pooled OAK and POPLAR trials.** Comparison of Clinical Benefit Between ICI and non-ICI Treatment for QoLS1(blue and lightblue) and QoLS2(red and pink) Groups in IMpower150 study(A); Comparison of Clinical Benefit Between ICI and non-ICI Treatment for QoLS1(blue and lightblue) and QoLS2(red and pink) Groups in the pooled OAK and POPLAR studies(B);

Abbreviations: CB, clinical benefit; NCB, No clinical benefit; ICI, Immune Checkpoint Inhibitor

## Supplementary Tables

| **Supplementary Table 1. OS-related subscales in HRQoL of discovery dataset** | | | | |
| --- | --- | --- | --- | --- |
| **EORTC QLQ-C30 V3** | |  | **EORTC QLQ-LC 13** | |
| **Subscale** | ***p*** |  | **Subscale** | ***p*** |
| Appetite loss symptom scale | 0.0000 |  | Alopecia | 0.4415 |
| Cognitive functional scale | 0.1647 |  | Coughing | 0.0014 |
| Constipation symptom scale | 0.0009 |  | Dysphagia | 0.0007 |
| Diarrhoea symptom scale | 0.6371 |  | Dyspnoea | <0.0001 |
| Dyspnoea symptom scale | <0.0001 |  | Haemoptysis | 0.0006 |
| Emotional functional scale | 0.0048 |  | Pain in arm or shoulder | 0.0279 |
| Fatigue symptom scale | <0.0001 |  | Pain in chest | 0.2072 |
| Financial difficulties symptom scale | 0.0098 |  | Pain in other parts | <0.0001 |
| Global health status scale | <0.0001 |  | Peripheral neuropathy | 0.9648 |
| Insomnia symptom scale | 0.0313 |  | Sore mouth | 0.1558 |
| Nausea and vomiting symptom scale | 0.0001 |  |  |  |
| Pain symptom scale | <0.0001 |  |  |  |
| Physical functional scale | <0.0001 |  |  |  |
| Role functional scale | <0.0001 |  |  |  |
| Social functional scale | <0.0001 |  |  |  |

| **Supplementary Table 2. Description of selected methods for HRQoL data** | | |
| --- | --- | --- |
| **Alogrithms** | **Data structure** | **Package** |
| CIMLR | List | CIMLR (V1.0.0) |
| NMF | Matrix | intNMF (V1.2.0) |
| ConsensusClustering | List | ConsensusClusterPlus( V1.64.0) |
| iCluster | Matrix | iClusterPlus (V1.36.1) |
| MCIA | List | omicade4 (V1.40.0) |
| PINSPLUS | List | PINSPlus (V2.0.7) |
| SNF | List of matrices | SNFtool (V 2.3.1) |
| MoCluster | List | mogsa (V1.34.0) |
| RGCCA | List | RGCCA (V 3.0.3) |
| iClusterBayes | Matrix | iClusterPlus (V1.36.1) |
| MixKernel | Matrix | mixKernel (V 0.9-1) |

| **Supplementary Table 3. Characteristics of patients in the discovery, and external validation cohorts** | | | | | |
| --- | --- | --- | --- | --- | --- |
| **Variable** | **ALL** | **BIRCH** | **IMpower150** | **OAK** | **POPLAR** |
|  | **n=2040** | **n=600** | **n=719** | **n=583** | **n=138** |
| **Age group** |  |  |  |  |  |
| <65 | 1095(53.8%) | 298(49.7%) | 393(54.7%) | 319(54.7%) | 85(61.6%) |
| >=65 | 940(46.2%) | 302(50.3%) | 321(45.0%) | 264(45.3%) | 53(38.4%) |
| **Sex** |  |  |  |  |  |
| Female | 799(39.3%) | 251(41.8%) | 276(38.7%) | 223(38.3%) | 49(35.5%) |
| Male | 1236(60.7%) | 349(58.2%) | 438(61.3%) | 360(61.7%) | 89(64.5%) |
| **Race** |  |  |  |  |  |
| Asian | 321(16.2%) | 74(12.5%) | 101(14.6%) | 123(21.9%) | 23(16.9%) |
| White | 1589(80.1%) | 500(84.3%) | 573(82.7%) | 411(73.3%) | 105(77.2%) |
| Other | 73(3.68%) | 19(3.20%) | 19(2.74%) | 27(4.81%) | 8(5.88%) |
| **ECOG-PS** |  |  |  |  |  |
| >=1 | 1259(62.0%) | 389(64.8%) | 412(57.9%) | 366(62.8%) | 92(67.6%) |
| 0 | 771(38.0%) | 211(35.2%) | 299(42.1%) | 217(37.2%) | 44(32.4%) |
| **Histology** |  |  |  |  |  |
| Non-squamous | 1662(81.8%) | 431(71.8%) | 711(100%) | 428(73.4%) | 92(66.7%) |
| Squamous | 370(18.2%) | 169(28.2%) | 0(0%) | 155(26.6%) | 46(33.3%) |
| **Smoking history** |  |  |  |  |  |
| Current | 328(16.1%) | 63(10.5%) | 163(22.8%) | 77(13.2%) | 25(18.1%) |
| Previous | 1333(65.5%) | 442(73.7%) | 405(56.7%) | 399(68.4%) | 87(63.0%) |
| Never | 374(18.4%) | 95(15.8%) | 146(20.4%) | 107(18.4%) | 26(18.8%) |
| **Liver metastasis** |  |  |  |  |  |
| Absent | 1698(83.4%) | 501(83.4%) | 624(87.4%) | 467(80.1%) | 106(76.8%) |
| Present | 337(16.6%) | 99(16.5%) | 90(12.6%) | 116(19.9%) | 32(23.2%) |
| **Number of metastasis** |  |  |  |  |  |
| <=3 | 1679(83.4%) | 488(84.4%) | 690(96.6%) | 404(69.3%) | 97(70.3%) |
| >3 | 334(16.6%) | 90(15.6%) | 24(3.36%) | 179(30.7%) | 41(29.7%) |
| **PD-L1** |  |  |  |  |  |
| Negative | 633(31.2%) | 0(0.00%) | 338(47.4%) | 246(42.6%) | 49(35.5%) |
| Positive | 1396(68.8%) | 600(100%) | 375(52.6%) | 332(57.4%) | 89(64.5%) |
| **QoLS** |  |  |  |  |  |
| QoLS1 | 1337(65.5%) | 392(65.3%) | 477(66.3%) | 379(65.0%) | 89(64.5%) |
| QoLS2 | 703(34.5%) | 208(34.7%) | 242(33.7%) | 204(35.0%) | 49(35.5%) |

| **Supplementary Table 4. Clustering performance evaluation** | | | | | |
| --- | --- | --- | --- | --- | --- |
| **Dataset** | **Method** | **K** | **Silhouette_Coefficient** | **Davies_Bouldin_Score** | **Calinski_Harabasz** |
| IMpower150.ICI | .CIMLR | K2 | 0.004622426 | 3.754359705 | 39.13687452 |
| IMpower150.ICI | .NMF | K2 | 0.276706612 | 1.651660113 | 262.7374573 |
| IMpower150.ICI | .ConsensusClustering | K2 | 0.222878652 | 1.745287258 | 229.9300475 |
| IMpower150.ICI | .iCluster | K2 | -7.7754E-05 | 27.10025456 | 0.967489026 |
| IMpower150.ICI | .PINSPlus | K2 | 0.02268526 | 2.1655209 | 80.53405654 |
| IMpower150.ICI | .SNF | K2 | -0.072227673 | 2.908541771 | 8.100845298 |
| IMpower150.ICI | .MoCluster | K2 | 0.199214425 | 2.304015174 | 89.76751108 |
| IMpower150.ICI | .MCIA | K2 | 0.163280919 | 2.867320005 | 21.20824881 |
| IMpower150.ICI | .RGCCA | K2 | 0.243538387 | 1.703796848 | 243.627433 |
| IMpower150.ICI | .iClusterBayes | K2 | 0.002613482 | 22.62518825 | 1.389261806 |
| IMpower150.ICI | .MixKernel | K2 | 0.116870898 | 3.031936679 | 69.05601009 |
| BIRCH | .CIMLR | K2 | -0.000836334 | 2.556109054 | 45.93529599 |
| BIRCH | .NMF | K2 | 0.28993906 | 1.598754466 | 215.2080439 |
| BIRCH | .ConsensusClustering | K2 | 0.218206112 | 1.737140419 | 177.6731854 |
| BIRCH | .iCluster | K2 | -2.41011E-06 | 26.3393248 | 0.796919216 |
| BIRCH | .PINSPlus | K2 | 0.06635551 | 2.206697644 | 79.19432555 |
| BIRCH | .SNF | K2 | -0.081922492 | 2.930626559 | 12.82105239 |
| BIRCH | .MoCluster | K2 | 0.180930803 | 2.354939651 | 95.151306 |
| BIRCH | .MCIA | K2 | 0.097849346 | 3.813668037 | 11.34613063 |
| BIRCH | .RGCCA | K2 | 0.29915772 | 1.665892832 | 166.7801557 |
| BIRCH | .iClusterBayes | K2 | -0.001429352 | 29.55127009 | 0.630777583 |
| BIRCH | .MixKernel | K2 | 0.020896142 | 3.475364381 | 33.5896585 |
| OAK.ICI | .CIMLR | K2 | 0.049597685 | 4.243958594 | 30.3700466 |
| OAK.ICI | .NMF | K2 | 0.271298407 | 1.647222211 | 202.1838979 |
| OAK.ICI | .ConsensusClustering | K2 | 0.198422231 | 1.758034853 | 163.9146497 |
| OAK.ICI | .iCluster | K2 | 0.004154651 | 16.30662157 | 2.061317859 |
| OAK.ICI | .PINSPlus | K2 | 0.062885135 | 2.058917281 | 79.91609998 |
| OAK.ICI | .SNF | K2 | -0.082193053 | 8.495788724 | 1.356044192 |
| OAK.ICI | .MoCluster | K2 | 0.232470829 | 2.019669498 | 105.3298436 |
| OAK.ICI | .MCIA | K2 | 0.108846021 | 3.786637319 | 28.71289296 |
| OAK.ICI | .RGCCA | K2 | 0.318528117 | 1.561776571 | 166.9020923 |
| OAK.ICI | .iClusterBayes | K2 | -0.000614313 | 25.48121934 | 0.84537181 |
| OAK.ICI | .MixKernel | K2 | 0.040702289 | 3.778106953 | 31.29355338 |
| POPLAR.ICI | .CIMLR | K2 | 0.077894539 | 3.67577685 | 8.192438885 |
| POPLAR.ICI | .NMF | K2 | 0.273351012 | 1.637485491 | 46.14342745 |
| POPLAR.ICI | .ConsensusClustering | K2 | 0.191874728 | 1.730304741 | 37.04906068 |
| POPLAR.ICI | .iCluster | K2 | 0.005366501 | 7.634440599 | 2.106180435 |
| POPLAR.ICI | .PINSPlus | K2 | 0.10277904 | 1.832561818 | 24.29477823 |
| POPLAR.ICI | .SNF | K2 | 0.001353056 | 2.587358855 | 9.062773177 |
| POPLAR.ICI | .MoCluster | K2 | 0.140964666 | 2.751644448 | 15.56992356 |
| POPLAR.ICI | .MCIA | K2 | 0.054696043 | 3.789807938 | 2.684942626 |
| POPLAR.ICI | .RGCCA | K2 | 0.303293461 | 1.577829231 | 45.20220866 |
| POPLAR.ICI | .iClusterBayes | K2 | 0.004075806 | 8.189194065 | 1.83184853 |
| POPLAR.ICI | .MixKernel | K2 | 0.055786894 | 3.715329565 | 8.259726855 |

| **Supplementary Table 5. Differences in Characteristics Between QOLS1 and QOLS2 in POPLAR** | | | |
| --- | --- | --- | --- |
| **Variable** | **QoLS1(n=89)** | **QoLS2(n=49)** | ***p*** |
| **AGE (mean (SD))** | 61.39 (9.68) | 61.18 (8.58) | 0.899 |
| **Age group (%)** |  |  |  |
| <65 | 55 ( 61.8) | 30 ( 61.2) | 1 |
| ≥65 | 34 ( 38.2) | 19 ( 38.8) |  |
| **SEX (%)** |  |  |  |
| Female | 31 ( 34.8) | 18 ( 36.7) | 0.97 |
| Male | 58 ( 65.2) | 31 ( 63.3) |  |
| **RACE (%)** |  |  |  |
| Asian | 20 ( 22.7) | 3 ( 6.2) | **0.033** |
| White | 63 ( 71.6) | 42 ( 87.5) |  |
| Other | 5 ( 5.7) | 3 ( 6.2) |  |
| **ECOG (%)** |  |  |  |
| 0 | 37 ( 42.5) | 7 ( 14.3) | **0.001** |
| ≥1 | 50 ( 57.5) | 42 ( 85.7) |  |
| **Smoking history (%)** |  |  |  |
| Current | 13 ( 14.6) | 12 ( 24.5) | 0.343 |
| Never | 18 ( 20.2) | 8 ( 16.3) |  |
| Previous | 58 ( 65.2) | 29 ( 59.2) |  |
| **Liver metastasis (%)** |  |  |  |
| Absent | 67 ( 75.3) | 39 ( 79.6) | 0.716 |
| Present | 22 ( 24.7) | 10 ( 20.4) |  |
| **metastasis3 (%)** |  |  |  |
| ≤3 | 65 ( 73.0) | 32 ( 65.3) | 0.45 |
| >3 | 24 ( 27.0) | 17 ( 34.7) |  |
| **PD-L1 (%)** |  |  |  |
| Negative | 32 ( 36.0) | 17 ( 34.7) | 1 |
| Positive | 57 ( 64.0) | 32 ( 65.3) |  |

| **Supplementary Table 6. Differences in Characteristics Between QOLS1 and QOLS2 in OAK** | | | |
| --- | --- | --- | --- |
| **Variable** | **QoLS1(n=379)** | **QoLS2(n=204)** | ***p*** |
| **AGE (median [IQR])** | 64.00 [57.00, 70.00] | 62.00 [55.00, 68.00] | 0.133 |
| **Age group (%)** |  |  |  |
| <65 | 200 ( 52.8) | 119 ( 58.3) | 0.23 |
| ≥65 | 179 ( 47.2) | 85 ( 41.7) |  |
| **SEX (%)** |  |  |  |
| Female | 142 ( 37.5) | 81 ( 39.7) | 0.659 |
| Male | 237 ( 62.5) | 123 ( 60.3) |  |
| **RACE (%)** |  |  |  |
| Asian | 93 ( 25.3) | 30 ( 15.5) | **0.025** |
| White | 258 ( 70.3) | 153 ( 78.9) |  |
| Other | 16 ( 4.4) | 11 ( 5.7) |  |
| **ECOG (%)** |  |  |  |
| 0 | 170 ( 44.9) | 47 ( 23.0) | **<0.001** |
| ≥1 | 209 ( 55.1) | 157 ( 77.0) |  |
| **Smoking history (%)** |  |  |  |
| Current | 50 ( 13.2) | 27 ( 13.2) | 0.938 |
| Never | 68 ( 17.9) | 39 ( 19.1) |  |
| Previous | 261 ( 68.9) | 138 ( 67.6) |  |
| **Liver metastasis (%)** |  |  |  |
| Absent | 319 ( 84.2) | 148 ( 72.5) | **0.001** |
| Present | 60 ( 15.8) | 56 ( 27.5) |  |
| **metastasis3 (%)** |  |  |  |
| ≤3 | 273 ( 72.0) | 131 ( 64.2) | 0.063 |
| >3 | 106 ( 28.0) | 73 ( 35.8) |  |
| **PD-L1 (%)** |  |  |  |
| Negative | 157 ( 41.5) | 89 ( 44.5) | 0.55 |
| Positive | 221 ( 58.5) | 111 ( 55.5) |  |

| **Supplementary Table 7. Univariable and Multivariable Cox Proportional Hazards Analysis for OS and PFS in OAK(treated with ICI).** | | | | | | | | | |
| --- | --- | --- | --- | --- | --- | --- | --- | --- | --- |
| **Variable** | **OS** | | | |  | **PFS** | | | |
|  | **Univariate HR(95%CI)** | ***p*** | **Multivariate HR(95%CI)** | ***p*** |  | **Univariate HR(95%CI)** | ***p*** | **Multivariate HR(95%CI)** | ***p*** |
| Age group |  |  |  |  |  |  |  |  |  |
| <65 | Reference |  | Reference |  |  | Reference |  | Reference |  |
| ≥65 | 0.94(0.77,1.16) | 0.5626 | 1(0.8,1.25) | 0.9924 |  | 1(0.84,1.19) | 0.9989 | 1.06(0.88,1.28) | 0.5276 |
| QoLS |  |  |  |  |  |  |  |  |  |
| QoLS1 | Reference |  | Reference |  |  | Reference |  | Reference |  |
| QoLS2 | 1.93(1.56,2.38) | **<0.001** | 1.56(1.24,1.95) | **0.0001** |  | 1.28(1.07,1.54) | **0.0064** | 1.12(0.92,1.36) | 0.2741 |
| ECOG PS |  |  |  |  |  |  |  |  |  |
| 0 |  |  |  |  |  |  |  |  |  |
| ≥1 | 1.81(1.45,2.25) | **<0.001** | 1.63(1.29,2.07) | **<0.001** |  | 1.23(1.03,1.47) | **0.0249** | 1.17(0.96,1.41) | 0.1136 |
| Number of metastatic |  |  |  |  |  |  |  |  |  |
| <=3 | Reference |  | Reference |  |  | Reference |  | Reference |  |
| >3 | 1.79(1.45,2.22) | **<0.001** | 1.23(0.92,1.63) | 0.1587 |  | 1.49(1.24,1.79) | **<0.001** | 1.19(0.93,1.52) | 0.1558 |
| PD-L1 |  |  |  |  |  |  |  |  |  |
| Positive | Reference |  | Reference |  |  | Reference |  | Reference |  |
| Negative | 1.2(0.98,1.48) | 0.0786 | 1.3(1.04,1.62) | **0.019** |  | 1.13(0.95,1.34) | 0.1756 | 1.1(0.92,1.32) | 0.2965 |
| Race |  |  |  |  |  |  |  |  |  |
| White | Reference |  | Reference |  |  | Reference |  | Reference |  |
| Asian | 0.7(0.53,0.92) | **0.0092** | 0.67(0.49,0.9) | **0.0092** |  | 1.09(0.88,1.34) | 0.4289 | 0.99(0.78,1.26) | 0.9312 |
| Other | 1.04(0.72,1.5) | 0.8318 | 0.88(0.59,1.31) | 0.5233 |  | 0.9(0.66,1.23) | 0.5094 | 0.85(0.6,1.19) | 0.3447 |
| Sex |  |  |  |  |  |  |  |  |  |
| Female | Reference |  | Reference |  |  | Reference |  | Reference |  |
| Male | 1.18(0.96,1.46) | 0.1219 | 1.25(0.98,1.59) | 0.0704 |  | 1.06(0.89,1.27) | 0.4983 | 1.18(0.96,1.45) | 0.1259 |
| Smoking history |  |  |  |  |  |  |  |  |  |
| Never | Reference |  | Reference |  |  | Reference |  | Reference |  |
| Previous | 1.14(0.92,1.43) | 0.2371 | 0.95(0.69,1.3) | 0.7398 |  | 0.96(0.8,1.16) | 0.6689 | 0.69(0.53,0.9) | **0.0053** |
| Current | 0.9(0.66,1.23) | 0.5153 | 0.88(0.57,1.36) | 0.5508 |  | 0.77(0.59,1) | 0.0524 | 0.58(0.4,0.83) | **0.0033** |

| **Supplementary Table 8. Univariable and Multivariable Cox Proportional Hazards Analysisfor OS and PFS in BIRCH.** | | | | | | | | | |
| --- | --- | --- | --- | --- | --- | --- | --- | --- | --- |
| **Variable** | **OS** | | | |  | **PFS** | | | |
|  | **Univariate HR(95%CI)** | ***p*** | **Multivariate HR(95%CI)** | ***p*** |  | **Univariate HR(95%CI)** | ***p*** | **Multivariate HR(95%CI)** | ***p*** |
| Age group |  |  |  |  |  |  |  |  |  |
| <65 | Reference |  | Reference |  |  | Reference |  | Reference |  |
| ≥65 | 0.85(0.65,1.12) | 0.2481 | 0.92(0.68,1.23) | 0.5658 |  | 0.92(0.77,1.11) | 0.3934 | 0.89(0.73,1.09) | 0.2729 |
| QoLS |  |  |  |  |  |  |  |  |  |
| QoLS1 | Reference |  | Reference |  |  | Reference |  | Reference |  |
| QoLS2 | 3.33(2.53,4.39) | **<0.001** | 2.94(2.15,4.01) | **<0.001** |  | 1.69(1.4,2.04) | **<0.001** | 1.53(1.23,1.9) | **0.0001** |
| ECOG PS |  |  |  |  |  |  |  |  |  |
| 0 | Reference |  | Reference |  |  | Reference |  | Reference |  |
| ≥1 | 2.06(1.51,2.82) | **<0.001** | 1.27(0.9,1.79) | 0.1793 |  | 1.28(1.06,1.56) | **0.0111** | 1.12(0.91,1.39) | 0.287 |
| Number of metastatic |  |  |  |  |  |  |  |  |  |
| ≤3 | Reference |  | Reference |  |  | Reference |  | Reference |  |
| >3 | 2.03(1.46,2.83) | **<0.001** | 1.26(0.84,1.87) | 0.2601 |  | 1.43(1.11,1.84) | **0.0051** | 1.15(0.85,1.56) | 0.3605 |
| Race |  |  |  |  |  |  |  |  |  |
| White | Reference |  | Reference |  |  | Reference |  | Reference |  |
| Asian | 1.16(0.77,1.75) | 0.4879 | 1.38(0.88,2.15) | 0.1612 |  | 1.26(0.96,1.66) | 0.0984 | 1.07(0.79,1.45) | 0.6589 |
| Other | 1.63(0.91,2.91) | 0.1033 | 1.75(0.91,3.35) | 0.0933 |  | 1.27(0.81,1.99) | 0.2957 | 1.49(0.9,2.45) | 0.1213 |
| Sex |  |  |  |  |  |  |  |  |  |
| Female | Reference |  | Reference |  |  | Reference |  | Reference |  |
| Male | 1.16(0.88,1.53) | 0.2958 | 1.28(0.95,1.73) | 0.1024 |  | 1.08(0.9,1.3) | 0.4193 | 1.25(1.02,1.53) | **0.0326** |
| Smoking history |  |  |  |  |  |  |  |  |  |
| Never | Reference |  | Reference |  |  | Reference |  | Reference |  |
| Previous | 0.88(0.65,1.2) | 0.4181 | 1.04(0.69,1.57) | 0.8382 |  | 0.8(0.65,0.98) | **0.0297** | 0.64(0.48,0.84) | **0.0013** |
| Current | 1.02(0.66,1.59) | 0.9305 | 1.03(0.58,1.86) | 0.91 |  | 0.8(0.58,1.1) | 0.1637 | 0.51(0.34,0.77) | **0.0013** |

| **Supplementary Table 9. Univariable and Multivariable Cox Proportional Hazards Analysis for OS and PFS in POPLAR(treated with ICI).** | | | | | | | | | |
| --- | --- | --- | --- | --- | --- | --- | --- | --- | --- |
| **Variable** | **OS** | | | |  | **PFS** | | | |
|  | **Univariate HR(95%CI)** | ***p*** | **Multivariate HR(95%CI)** | ***p*** |  | **Univariate HR(95%CI)** | ***p*** | **Multivariate HR(95%CI)** | ***p*** |
| Age group |  |  |  |  |  |  |  |  |  |
| <65 | Reference |  | Reference |  |  | Reference |  | Reference |  |
| >=65 | 0.94(0.59,1.51) | 0.8075 | 1.28(0.72,2.3) | 0.4005 |  | 0.83(0.57,1.22) | 0.3457 | 0.95(0.61,1.49) | 0.826 |
| QoLS |  |  |  |  |  |  |  |  |  |
| QoLS1 | Reference |  | Reference |  |  | Reference |  | Reference |  |
| QoLS2 | 2.75(1.74,4.35) | **<0.001** | 3(1.69,5.29) | **0.0002** |  | 1.24(0.85,1.81) | 0.2609 | 1.18(0.75,1.85) | 0.481 |
| ECOG PS |  |  |  |  |  |  |  |  |  |
| 0 | Reference |  | Reference |  |  | Reference |  | Reference |  |
| >=1 | 1.64(0.97,2.76) | 0.0646 | 0.77(0.38,1.56) | 0.4635 |  | 1.53(1.02,2.27) | **0.0376** | 1.37(0.83,2.27) | 0.2184 |
| Number of metastatic |  |  |  |  |  |  |  |  |  |
| <=3 | Reference |  | Reference |  |  | Reference |  | Reference |  |
| >3 | 2.19(1.36,3.51) | **0.0012** | 1.78(0.95,3.33) | 0.0714 |  | 1.81(1.23,2.68) | **0.0028** | 1.13(0.69,1.85) | 0.6307 |
| PD-L1 |  |  |  |  |  |  |  |  |  |
| Positive | Reference |  | Reference |  |  | Reference |  | Reference |  |
| Negative | 1.6(1.01,2.55) | **0.0461** | 1.42(0.84,2.4) | 0.1866 |  | 1.24(0.85,1.81) | 0.2699 | 1.45(0.95,2.21) | 0.0882 |
| Race |  |  |  |  |  |  |  |  |  |
| White | Reference |  | Reference |  |  | Reference |  | Reference |  |
| Asian | 0.45(0.21,0.99) | **0.0475** | 0.73(0.29,1.85) | 0.507 |  | 1.06(0.65,1.74) | 0.8184 | 1.21(0.64,2.31) | 0.5523 |
| Other | 1.24(0.54,2.86) | 0.6117 | 0.6(0.22,1.65) | 0.3188 |  | 0.99(0.5,1.95) | 0.9665 | 0.66(0.3,1.48) | 0.3138 |
| Sex |  |  |  |  |  |  |  |  |  |
| Female | Reference |  | Reference |  |  | Reference |  | Reference |  |
| Male | 1.18(0.72,1.92) | 0.5109 | 1.75(0.91,3.36) | 0.096 |  | 0.91(0.63,1.33) | 0.6386 | 0.82(0.5,1.33) | 0.4121 |
| Smoking history |  |  |  |  |  |  |  |  |  |
| Never | Reference |  | Reference |  |  | Reference |  | Reference |  |
| Previous | 1.3(0.81,2.11) | 0.2808 | 1.6(0.68,3.75) | 0.2816 |  | 1.14(0.78,1.65) | 0.5047 | 1(0.57,1.78) | 0.9887 |
| Current | 1.21(0.7,2.11) | 0.4973 | 1.53(0.57,4.1) | 0.3964 |  | 0.7(0.43,1.13) | 0.1433 | 0.65(0.31,1.38) | 0.2625 |
